# Micro‐sub regional synapse weakening by mimicking the hyperphosphorylation of microtubule associated protein Tau in dendritic spines

**DOI:** 10.1002/alz70855_102144

**Published:** 2025-12-23

**Authors:** Kei Kwangwook Cho

**Affiliations:** ^1^ King's College London, London, London, United Kingdom

## Abstract

**Background:**

The aberrant phosphorylation of Tau is a phenotype of many neurodegenerative disorders which is suggested to be a causal pathological feature of cognitive impairment. We have previously illustrated the importance of specific tau phosphorylation sites (396/404) in mediated synapse weakening, and the interplay of the Tau interactome, specifically PACSIN1 in the process. However, a knowledge gap exists in understanding how pTau induced pathophysiology progresses, especially considering synapse dysfunction can occur without total neuronal atrophy. This opens the possibility that the small dendritic regions could undergo synapse dysfunction and underpin wider cognitive impairments.

**Method:**

Utilising organotypic hippocampal slice culture with biolistic transfection, we express human full‐length Tau phosphomimic (TauPHF1E) and phosphonull (TauPHF1A) constructs in CA1 neurons. For all experiments we utilised multiphoton or spinning disk confocal imaging of a distal and proximal region of the same secondary dendrite. Individual dendritic spines were stimulated via glutamate uncaging and plasticity (spine area change) monitored over time. Subsequently Fibronetic intrabody display PSD‐95(GFP) was coexpressed to allow for endogenous PSD‐95 expression and dynamics to be examined.

**Result:**

We analysed activity‐dependent single dendritic spine plasticity in the proximal and distal dendritic regions which were stimulated via two‐photon glutamate uncaging. We observed a TauPHF1E induced impairment of structural plasticity only in the distal dendritic region, conversely the proximal region of the same dendrite exhibited normal structural plasticity. Subsequently, we analysed the expression and dynamics of PSD‐95. TauPHF1E reduced PSD‐95 puncta number and impaired its mobility as observed by fluorescent recovery after bleaching. As we have previously observed that pTau‐mediated synapse weakening requires PACSIN1, we co‐expressed shRNA PACSIN in addition to TauPHF1E. The reduction of PACSIN1 rescued the observed synapse weakening deficits in the distal dendritic region.

**Conclusion:**

Here, we illustrate, that Tau‐PHF1E, a human tau phosphomimic construct, exhibited a spatially distribution of pathophysiology, with the distal dendric regions of stratum radiatum exhibiting dysfunction while the proximal region of the same dendrites remained unaffected. Furthermore, we observed that these deficits were dependent upon the presence of a key tau interacting protein; protein kinase C and casein kinase substrate in neurons protein 1 (PACSIN1).

